# Prevalence and gender disparities of type 2 diabetes mellitus and obesity in Esmeraldas, Ecuador: a population-based survey in a hard-to-reach setting

**DOI:** 10.1186/s12939-023-01939-x

**Published:** 2023-07-01

**Authors:** Marta Puig-García, Cintia Caicedo-Montaño, Mónica Márquez-Figueroa, Elisa Chilet-Rosell, Gregorio Montalvo-Villacis, Ikram Benazizi-Dahbi, Andrés Peralta, Ana Lucía Torres-Castillo, Lucy Anne Parker

**Affiliations:** 1 Department of Public Health, Universidad Miguel Hernández de Elche, Alicante, Spain; 2 CIBER de Epidemiología y Salud Pública (CIBERESP), Madrid, Spain; 3 Centre of Community Epidemiology and Tropical Medicine (CECOMET), Esmeraldas, Ecuador; 4 School of Medical Specialities, Colegio de Ciencias de la Salud, Universidad San Francisco de Quito, Quito, Ecuador; 5 Institute of Public Health, Faculty of Medicine, Pontificia Universidad Católica del Ecuador, Quito, Ecuador

**Keywords:** Diabetes mellitus, Obesity, Health disparity, Social determinants of health, Cross-sectional study, Ecuador

## Abstract

**Background:**

Type 2 Diabetes (T2DM) prevalence is increasing in low- and middle-income countries along with high levels of obesity which vary according to socioeconomic and contextual characteristics. We aim to estimate the prevalence of T2DM and obesity in men and women in a hard-to-reach rural area in northern Ecuador considering socio-demographic characteristics.

**Methods:**

Cross-sectional descriptive study based on a population-based survey in the Eloy Alfaro health district of Esmeraldas between October 2020 and January 2022. We collected sociodemographic information and risk factors for non-communicable diseases with an adapted version of the STEPS survey, performed oral glucose tolerance tests, biochemistry and took physical measurements. We estimated the prevalence of T2DM, obesity, and calculated Odds Ratios (OR) with confidence intervals by logistic regression in Stata v.15.

**Results:**

The overall prevalence of T2DM was 6.8% (CI95%: 4.9–8.7), markedly higher among women compared to men (10.4%, CI95% 7.3–13.4%, compared 2.0%, CI95% 0.4–3.7% respectively). The risk of having T2DM in women was 5 times higher than in men adjusting for age, ethnicity, employment, household earnings and obesity (OR: 5.03; 95%CI: 1.68–15.01). Regarding age, the risk of T2DM increased by 6% per year (adjusted OR: 1.06; 95%CI: 1.03–1.08). Obesity prevalence was 30.8% (CI95%: 27.3–34.3), in women was nearly three times higher than in men (43.2% CI95%: 38.2–48.2, compared to 14.7% prevalence, CI95%: 10.6–18.8). Indigenous women had a lower prevalence of obesity compared with the Afro-Ecuadorian women (OR: 0.05; 95%CI: 0.02–0.18) after adjusting for age, employment status, household earnings and setting.

**Conclusion:**

We found alarming differences between the prevalence of T2DM and obesity in women and men that may be explained by gender roles, exacerbated in the rural context. Health promotion measures with a gender perspective should be adapted according to the characteristics of isolated rural contexts.

## Background

Type 2 Diabetes mellitus (T2DM) is a major public health problem due to its increasing prevalence, high morbidity and mortality, and high healthcare costs. Although the disease now affects more than 500 million people, low- and middle-income countries are the most affected and already have four-fifths of the population with diabetes [1]. Moreover, prevalence is expected to continue to increase rapidly (21% in middle-income countries) until 2045 due to population ageing [2]. Another driver of non-communicable diseases (NCDs), including diabetes, is rapid urbanisation and facilitated access to tobacco, alcohol and unhealthy foods, which create an obesogenic environment. Exposure to these environmental risk factors is leading to worrying levels of obesity in regions of Latin America and the Caribbean (LAC), where the prevalence is already 24% [3].

According to the CARMELA study, individuals with obesity are two to three times more likely to have non-communicable diseases (NCDs) such as type 2 diabetes (T2DM), hypertension, and dyslipidaemia [3]. With 63% of the Ecuadorian population being overweight or obese, this indicates a significant increase in T2DM cases in the future [4]. The International Diabetes Federation (IDF) 2021 report estimates that 4.7% (4.0–7.3) of the Ecuadorian population aged 20–79 years already has T2DM [2]. In 2021, diabetes was the second leading cause of death in Ecuador, excluding COVID deaths [5]. In a country with a 40% poverty rate, fragmented health systems and high out-of-pocket health costs, diabetes-related expenditures per person are estimated at approximately 2,280 (USD) per year and can place a significant financial burden on individuals for effective disease management [2, 6].

Although diabetes and obesity affect all age groups, there are socioeconomic and contextual differences that influence disease risk, such as ethnicity, gender, region or income [7, 8]. Several studies report differences in the prevalence of diabetes between men and women [1, 4, 8–10], which, as with other diseases, are determined by both sex, biological differences, and gender, predominant psychosocial influences [9]. Both factors interrelate and interact throughout people's lives, so when we talk about gender inequalities, we are referring to how gender (understood as culture-bound conventions, roles, and behaviours for, as well as relationships between and among, women and men) influences existing differences in the development of the disease [11]. The aim of the study is to estimate the prevalence of T2DM and obesity in men and women in a hard-to-reach rural area in northern Ecuador, taking into account sociodemographic characteristics.

## Methods

### Study design

Population-based cross-sectional study conducted in the Eloy Alfaro health district of Esmeraldas Province, Ecuador.

### Setting

The survey was conducted between October 2020 and January 2022 in the health district 08D02 (Eloy Alfaro) in Esmeraldas Province, a densely forested rural area in the northwest coastal region of the country with a population of 45,629 (est. 2020) [12]. Different ethnic groups coexist in this region, where approximately 85% of the population self-identify as afro-Ecuadorian, 10% as indigenous Chachi and 5% as mestizo (person of mixed ethnic heritage, e.g. European and Indigenous American; European and Afro-Ecuadorian; Indigenous American and Afro-Ecuadorian). The main economic activities in the area are dedicated to livestock, agriculture, fishing and/or tourism. The majority of the population (84%) live in small communities (some of which are only accessible by water) lining the Santiago, Cayapas and Onzole rivers, and 16% live in two semi-urban nuclei, Limones and Borbón. Esmeraldas is one of the provinces most affected by poverty and inequality in Ecuador [13]. In 2022, the unemployment rate was 9.1%, and the rate of poverty due to unsatisfied basic needs was 52.3% [14].

### Participants

As described in the study protocol, we proposed a sample size of 720 individuals [15] assuming the prevalence of T2DM is no higher than 10%, a design effect of 1.5 and a possible 20% loss of participants. All Eloy Alfaro residents aged over 18 years who provided informed consent were eligible for inclusion in the survey. Individuals were considered residents if they had slept in the district at least 20 days of the previous month and had no plan to move in the near future. The sampling procedures depended on the context with geospatial sampling being used for the urban centres and multistage stratified cluster sampling for rural communities. We aimed to recruit 240 individuals in the urban centres by selecting 328 randomly generated GPS points with QGIS Geographic Information System (QGIS Association. http://www.qgis.org) from the residential portions of the census sections in the urban centres (207 in Borbón and 121 in Limones). Community health promotors engaged by the Centre of Community Epidemiology and Tropical Medicine (CECOMET) visited the closest house to each GPS point and listed all individuals that resided within the house. We randomly selected one individual from the list and if they were not present when the survey team visited, the survey team tried to reschedule their visit up to 3 times, after which the GPS point was replaced. At times there was a significant time lag from listing the residents of the house to inviting the selected individual to participate in the study. The survey team visited new GPS points until the proposed sample size was achieved. We intended to recruit 480 individuals from the 150 rural communities by multistage stratified cluster sampling. Firstly, each community was classified according to the majority ethnicity (afro-Ecuadorian, indigenous, mestizo or mixed) and isolation (three categories according to the distance and time from the main urban area following the riverbeds) to define a total of 12 strata. We then selected 60 community clusters, whereby a number of communities were selected in each stratum with disproportionate allocation (i.e., number of communities proportionate to the population estimate in that stratum). Finally, from each cluster, we selected 8 participants using simple random sampling which was possible thanks to an existing census developed and updated between November 2018 and January 2020 by community health promotors in collaboration with CECOMET.

### Data collection

The survey team was made up of 4 women and 4 men aged between 24 and 57 from different cultural and ethnic groups, all with healthcare training (nurses, auxiliary nurses, midwifery). At least one member of the team was able to translate questions verbally to Cha’palaa (the language spoken by the indigenous population in the area) when required. We collected information on sociodemographic characteristics and health-related behaviours through face-to-face questionnaires in the participants’ homes. We used the WHO STEPS NCD risk factor survey forms [16], composed of core questions that obtain general information about risk factors and extended questions, to complement the first ones with more detailed information, with cultural adaptations to the questions as required. We included core and extended questions on education, ethnicity, marital status, employment status, household income, tobacco use and physical activity. Only the core questions were used to collect information about alcohol consumption and diet. After the interview, the survey team then arranged an appointment for physical and biological measurements together with the participant, considering geographical convenience and availability. At this second appointment, we measured weight, height, waist circumference and blood pressure according to the technical specifications recommended in the STEPS guideline [17]. Participants were instructed to fast for at least 8 h prior to the appointment. In order to determine eligibility for an Oral Glucose Tolerance Test (OGTT), we obtained a first blood sample from the study participants in a well-lit and hygienic environment. This initial blood sample was analysed using a capillary blood glucose test. Individuals with normal capillary glucose levels (< 144 mg/dl) were selected to undertake the OGTT, which involved a second blood draw 2 h after the ingestion of 75 g glucose (GLUTEST 75 g—DQGLT-075–001 – Quimical EC, Quito, Ecuador). Participants with diabetes or pregnant women were eligible for the study but did not undergo the OGTT. We offered a pregnancy test to women who were unsure of their pregnancy status. Samples were centrifuged using a mobile lab after being stored at -20ºC until analysis (average time 2 days) by a laboratory with international certification ISO 9001 (LABORATORIO CENTRO MÉDICO “MADRE ANASTASIA”). In the rural communities where the cold chain was not assured, samples were stored at -195ºC using liquid nitrogen (66 individuals, 132 samples). Information was recorded on digital tablets (Samsung Galaxy Tab AT290) with the programme KoboCollect (version 2.4).

### Variables

The main outcome variables of this study are type 2 diabetes and obesity. The diagnosis of T2DM was primarily made using the OGTT (2-h blood glucose values ≥ 200 mg/dl) as it is considered the gold standard for diagnosing T2DM due to its higher sensitivity according to the IDF guidelines [18]. For individuals who did not undergo an OGTT, we based the diagnosis of T2DM on their fasting plasma glucose levels (≥ 126 mg/dl). We calculated the body mass index (BMI) of the individuals and obesity was classified when BMI was equal to or higher than 30.

We categorised the following sociodemographic and behavioural variables to facilitate the analysis and interpretation. Age was considered as a continuous variable and when required categorised into 3 groups (18–39 years, 40–64 years, and ≥ 65 years). Self-reported ethnicity was simplified to Afro-Ecuadorian, Mestizo and Indigenous. We classified education into three levels (no formal schooling, primary school completed and secondary school completed or higher). Marital status was classified into two categories: partnered (married or free union) or unpartnered (single, separated or widow). Employment status was dichotomised into formal employment (including self-employed, private sector employee or government employee) or not in formal employment (homemakers, students, unemployed or retired) due to the low frequencies in some categories. The item for estimated household earnings was divided according to the median ($0 to $100 and over $100). Tobacco consumption was classified into three categories (never smoker, ex-smoker, smoker). Alcohol consumption was calculated based on the reported frequency and quantity of alcohol intake and classified as low-risk consumption when individuals consumed less than 60 g/day for men and 40 g/day for women. Physical activity included both recreational activities and work and is reported according to the WHO recommendation of at least 150 min of moderate to high-intensity activity per week. We considered diets to be unhealthy considering consumption of fruit and vegetables (< 2 portions/day), salt (always adding salt to food and eating processed food 5 or more times per week) and sugar (always adding sugar to beverages or consuming sweets or sweetened drinks 5 or more times per week). Although the recommended fruit and vegetable consumption is 5 pieces per day, we used 2 pieces as a reference due to the low fruit and vegetable consumption in this population.

### Data analysis

We conducted all statistical analyses using Stata version 15.0 (StataCorp, College Station, TX, USA). We described the prevalence of our main outcome variables with 95% confidence intervals, disaggregated by sex. We compared the sociodemographic and behavioural characteristics using proportions and Fisher’s exact test for categorical variables, and the mean and Student’s t-test for continuous variables. We used logistic regression to estimate Odds Ratios (ORs) with 95% confidence intervals and created a multivariable model to assess the role of sex in diabetes and obesity (separately) using backward elimination considering all variables that are known risk factors for diabetes or obesity, were associated with the outcome variable (diabetes or obesity) in the univariate analysis with at least a p-value ≤ 0.1 or showed potentially meaningful differences between men and women. Missing data were excluded from the logistic regression analysis. We considered confounding to be present when the OR for sex varied by at least 10% after removing the variable from the model. The factors associated with obesity in men and women are reported separately due to the differences observed (effect modification). We considered the pertinence of performing the analysis segregated by ethnicity due to marked cultural differences relating to living and working conditions among the indigenous population. We performed a sensitivity analysis excluding the indigenous population and found no impact in the findings presented here. Thus, a global analysis is presented where ethnicity is included as a potential explanatory variable. All analyses considered the sampling strategy used in the survey and were performed by sex to facilitate the gender analysis in the interpretation of the results. Missing data were excluded from the models.

## Results

**Fig. 1:**
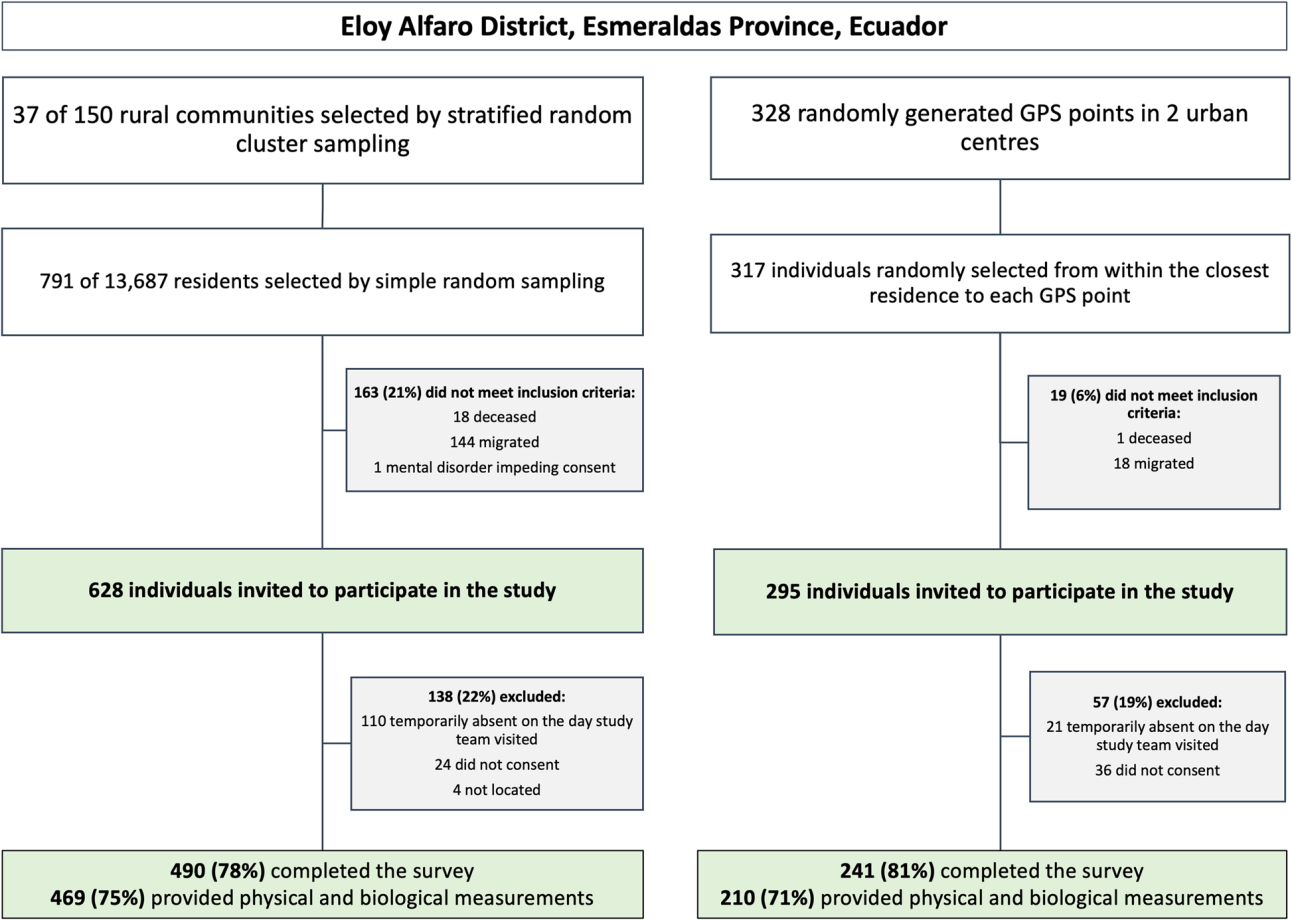
Participant flow diagram

We randomly selected 791 individuals from the total of 13,687 residents living in 150 rural communities in Eloy Alfaro from which 490 completed the survey (78% of those who met the inclusion criteria) and 469 also completed physical and biological measurements. In the urban communities of Borbón and Limones, 241 (81%) completed the survey and 210 (71%) provided physical and biological measurements (Fig. 1).

Table 1 presents the baseline characteristics of the study population. In total, 731 people completed the population survey, of whom 407 were women (55.5%), and 680 also completed the physical and biochemical measurements (386 women, 56.8%). The mean age for men was 44 years (range 19–88 years) and 42 years for women (range 18–93). Regarding ethnicity, the majority of the participants were Afro-Ecuadorian. Except for education level, which was similar between men and women with approximately one-fourth of the population without formal schooling, significant differences in sociodemographic items (employment status, household earnings and setting) were found between men and women: a higher percentage of women had no formal employment and reported household earnings of less than $100, compared to men. Residing in a rural setting was more frequent among men than women.

**Table 1:** Baseline characteristics of the study population (*n* = 731)

	**Men**		**Women**		**Total**		***p*-value**
	N	(%)	N	(%)	N	(%)	
**Sociodemographic factors**							
** Age in years, mean (SD)**	44	(± 0.9)	42	(± 0.8)	43	(± 0.6)	0.069
** Ethnicity**							0.096
Afro	191	(58.8)	231	(56.9)	422	(57.7)	
Mestizo	77	(23.7)	121	(29.8)	198	(27.1)	
Indigenous	57	(17.5)	54	(13.3)	111	(15.2)	
** Education level**^a^							0.883
No formal schooling	73	(22.5)	90	(22.2)	163	(22.3)	
Primary school	122	(37.5)	159	(39.3)	281	(38.5)	
Secondary school or higher	130	(40.0)	156	(38.5)	286	(39.2)	
** Marital status**							0.288
In a relationship	237	(72.9)	281	(69.2)	518	(70.9)	
Unpartnered	88	(27.1)	125	(30.8)	213	(29.1)	
** Employment status**							< 0.001
Unemployed	30	(9.2)	298	(73.4)	328	(44.9)	
Employed	295	(90.8)	108	(26.6)	403	(55.1)	
** Household earnings**^b^							0.003
≤ 100$	149	(49.7)	226	(61.2)	375	(56.1)	
> 100$	151	(50.3)	143	(38.8)	294	(43.9)	
** Setting**							0.027
Rural	232	(71.4)	258	(63.5)	490	(67.0)	
Urban	93	(28.6)	148	(36.5)	241	(33.0)	
**Behavioural factors**							
** Tobacco consumption**							< 0.001
Never smoker	225	(69.2)	357	(87.9)	582	(79.6)	
Ex-smoker	41	(12.6)	23	(5.7)	64	(8.8)	
Smoker	59	(18.2)	26	(6.4)	85	(11.6)	
** Alcohol consumption**							< 0.001
Low risk	245	(75.4)	355	(87.4)	600	(82.1)	
High risk	80	(24.6)	51	(12.6)	131	(17.9)	
** Physical activity**							< 0.001
≥ 150 min/week	298	(91.7)	318	(78.3)	616	(84.3)	
< 150 min/week	27	(8.3)	88	(21.7)	115	(15.7)	
** Diet**							1.000
Healthy	56	(17.2)	70	(17.2)	126	(17.2)	
Unhealthy	269	(82.8)	336	(82.8)	605	(82.8)	

^a^1 missing education

^b^39 do not know, 23 do not answer income

There were also important differences regarding certain behavioural items. Men had a higher frequency of tobacco and alcohol consumption, while women wore frequently failed to meet the physical activity recommendations. Regarding diet, 605 individuals (82.8%) reported unhealthy diets, where low consumption of fruit and vegetables stands out, with more than three in four individuals having less than two portions (532 individuals, 73%, showing no differences by sex (data not shown in table)). Two out of five people reported consumption of sweets or sweetened drinks 5 or more days per week (40.5%), contrasting with the low consumption of processed foods high in salt (3%). Men answered always adding salt to foods (19.7%) more frequently than women (12.8%), while there was no difference in the addition of sugar to beverages (37%) (data not shown in table).

### Diabetes prevalence and associated factors

Forty-six participants had T2DM (47.8% were unaware of their condition at study recruitment), giving an overall prevalence of 6.8% (CI95%: 4.9–8.7). The prevalence was markedly higher among women compared to men (40 women, 10.4% prevalence, CI95% 7.3–13.4%, compared to 6 men, 2.0% prevalence, CI95% 0.4–3.7%). The risk of having T2DM in women was 5 times higher than in men adjusting for age, ethnicity, employment, household earnings and BMI (OR: 5.03; 95%CI: 1.68–15.01, Table 2). Age was also associated with an increased risk of T2DM by 6% per year (adjusted OR: 1.06; 95%CI: 1.03–1.08), and the risk of developing T2DM was 3.46 times higher in people with obesity (BMI ≥ 30) (adjusted OR: 2.63; 95%CI: 1.03–6.73). Compared with the Afro-Ecuadorian, the Indigenous ethnicity was a protective factor against T2DM (OR: 0.1; 95%CI: 0.01–0.74). Also, individuals in formal employment showed a reduction in the risk of T2DM by 57% (OR: 0.43; 95CI%: 0.23–0.80). However, both ethnicity and employment status lost significance once adjusted in the model with the rest of factors. Education, marital status, household income and setting were not associated with T2DM.

**Table 2:** Diabetes prevalence and associated factors in the study population of Esmeraldas

	N (%)		Total	*p* value^*^	OR	95% CI	*p*-value	ORa^a^	95% CI	*p*-value
Sex				** < 0.001**						
Men	6	(2.0)	293		1			1		
Women	40	(10.4)	386		5.53	[2.31- 13.23]	** < 0.001**	5.03	[1.68- 15.02]	**0.004**
Age in years, mean (SD)	55 ± 2		43 ± 1	** < 0.001**	1.04	[1.02- 1.06]	** < 0.001**	1.06	[1.03- 1.08]	** < 0.001**
Ethnicity				**0.008**						
Afro	34	(8.7)	393		1			1		
Mestizo	11	(6.2)	179		0.69	[0.34- 1.40]	0.304	0.69	[0.32- 1.48]	0.341
Indigenous	1	(0.9)	107		0.10	[0.01- 0.74]	**0.024**	0.24	[0.03- 1.87]	0.172
Education level^b^				0.273						
No formal schooling	14	(8.9)	157		1					
Primary school	19	(7.3)	262		0.80	[0.39- 1.64]	0.541			
Secondary school or higher	13	(5.0)	259		0.54	[0.25- 1.18]	0.123			
Marital status				1.000						
In a relationship	33	(6.9)	479		1					
Unpartnered	13	(6.5)	200		0.94	[0.48- 1.83]	0.854			
Employment status				**0.009**						
Unemployed	30	(9.6)	312		1			1		
Employed	16	(4.3)	369		0.43	[0.23- 0.80]	**0.008**	0.70	[0.32- 1.57]	0.391
Household earnings^c^				0.750						
≤ 100$	26	(7.3)	356		1			1		
> 100$	17	(6.4)	265		0.87	[0.46- 1.64]	0.665	1.24	[0.59- 2.61]	0.579
Setting				0.408						
Rural	29	(6.2)	469		1					
Urban	17	(8.1)	210		1.34	[0.72- 2.49]	0.367			
BMI^d^				**0.001**						
18.5–24.9	9	(4.0)	227		1			1		
25.0–29.9	11	(5.0)	221		1.27	[0.52- 3.12]	0.605	1.13	[0.41- 3.10]	0.814
≥ 30.0	26	(12.5)	208		3.46	[1.58- 7.57]	**0.002**	2.63	[1.03- 6.73]	**0.044**
Total^e^	46	(6.8)	679							

^*^p-value < 0.05 (t-student for age and Fisher’s exact test for the rest)

^a^Adjusted by sex, age, ethnicity, employment, household earnings and BMI

^b^One not reported education

^c^38 do not know, 20 do not answer income

^d^18 cases were excluded with a BMI under 18.5 and five cases had no BMI due to pregnancy

^e^*N* = 679 completed biological measurements

### Obesity prevalence and associated factors

Two hundred and eight individuals were classified as obese, giving a global prevalence of 30.8% (CI95%: 27.3–34.3). The prevalence of obesity in women was three times higher than in men (165 women, 43.2% prevalence, CI95%: 38.2–48.2, compared to 43 men, 14.7% prevalence, CI95%: 10.6–18.8). The prevalence of obesity among women under the age of 65 was particularly high, with over half of the women aged between 40 and 64 years old being obese and 42.4% of younger women aged 18–39 (Table 3). This contrasted with men, where there were no marked differences in obesity in the different age groups (Table 4). Furthermore, among men, none of the socioeconomic or cultural factors studied appeared to be associated with obesity. Among women, there were differences in the prevalence of obesity according to age, ethnicity, employment status and setting. However, after conducting multivariable logistic regression analyses, only age and ethnicity remained significant, showing that indigenous women had a significantly lower prevalence of obesity compared with the Afro-Ecuadorian women (OR: 0.05; 95%CI: 0.02–0.18) after adjusting for age, employment status, household earnings and setting. Although not significant, data show that men with formal jobs, higher incomes and from urban areas may have higher levels of obesity.

**Table 3:** Obesity prevalence and associated factors in women in the study population of Esmeraldas

	N (%)		Total	*p* value^*^	OR	95% CI	*p*-value	ORa^a^	95% CI	*p*-value
Age in years				**0.003**						
18–39	84	(42.4)	198		1			1		
40–64	73	(50.3)	145		1.4	[0.89- 2.12]	0.15	1.25	[0.76- 2.06]	0.376
≥ 65	8	(20.5)	39		0.35	[0.15- 0.80]	**0.013**	0.22	[0.09- 0.55]	**0.001**
Ethnicity				** < 0.001**						
Afro	114	(50.9)	224		1			1		
Mestizo	48	(44.4)	108		0.77	[0.49- 1.22]	0.271	0.92	[0.55- 1.53]	0.752
Indigenous	3	(6.0)	50		0.06	[0.02- 0.20]	** < 0.001**	0.05	[0.02- 0.18]	** < 0.001**
Education level^b^				0.432						
No formal schooling	33	(37.5)	88		1					
Primary school	69	(46.6)	148		1.46	[0.83- 2.45]	0.172			
Secondary school or higher	62	(42.8)	145		1.25	[0.72- 2.14]	0.429			
Marital status				0.434						
In a relationship	118	(44.7)	264		1					
Unpartnered	47	(39.8)	118		0.82	[0.53- 1.27]	0.375			
Employment status				**0.080**						
Unemployed	113	(40.4)	280		1					
Employed	52	(51.0)	102		1.54	[0.97- 2.42]	**0.065**	1.27	[0.74- 2.21]	0.388
Household earnings^c^				0.435						
≤ 100$	89	(41.8)	213		1					
> 100$	62	(46.6)	133		1.22	[0.79- 1.89]	0.378	0.71	[0.43- 1.18]	0.186
Setting				**0.040**						
Rural	97	(39.3)	247		1					
Urban	68	(50.4)	135		1.57	[1.03- 2.40]	**0.037**	1	[0.62- 1.62]	0.993
Total^d^	165	(43.2)	382							

^*^p-value < 0.05 (t-student for age and Fisher’s exact test for the rest)

^a^Adjusted by age, ethnicity, employment status, household earnings and setting

^b^One not reported education

^c^22 do not know, 14 do not answer income

^d^5 women were not included

**Table 4:** Obesity prevalence and associated factors in men in the study population of Esmeraldas

	N (%)		Total	p value^*^	OR	95% CI	p-value
Age in years				0.898			
18–39	18	(14.2)	127		1		
40–64	20	(15.9)	126		1.14	[0.57- 2.28]	0.705
≥ 65	5	(12.5)	40		0.87	[0.30- 2.50]	0.789
Ethnicity				0.166			
Afro	26	(15.4)	169		1		
Mestizo	13	(18.8)	69		1.28	[0.61- 2.66]	0.514
Indigenous	4	(7.3)	55		0.43	[0.14- 1.30]	0.134
Education level				0.955			
No formal schooling	11	(15.9)	69		1		
Primary school	16	(14.3)	112		0.88	[0.38- 2.02]	0.761
Secondary school or higher	16	(14.3)	112		0.88	[0.38- 2.02]	0.761
Marital status				0.358			
In a relationship	34	(16.1)	211		1		
Unpartnered	9	(11.0)	82		0.64	[0.29- 1.41]	0.267
Employment status				0.393			
Unemployed	2	(7.4)	27		1		
Employed	41	(15.4)	266		2.27	[0.52- 9.94]	0.278
Household earnings^a^				0.211			
≤ 100$	15	(10.6)	141		1		
> 100$	21	(16.2)	130		1.62	[0.80- 3.29]	0.184
Setting				0.129			
Rural	28	(12.8)	219		1		
Urban	15	(20.3)	74		1.73	[0.87- 3.47]	0.119
Total	43	(14.6)	293				

^*^p-value < 0.05 (t-student for age and Fisher’s exact test for the rest)

^a^16 do not know, 6 do not answer income

## Discussion

Our study shows important differences in the prevalence of diabetes and obesity between men and women in this rural area of Ecuador. Perhaps the most striking finding is that women in the Eloy Alfaro district are five times more likely to have diabetes than men, reaching a prevalence of 10.4%. A national survey from 2012 in Ecuador showed an overall prevalence in the country of 2.7% (CI95% 2.2–3.3%) with barely any difference between men (2.6%) and women (2.8%) [4]. Additionally, while the indigenous ethnic group has a similar low prevalence of diabetes in both studies, the percentage in the Afro-Ecuadorian community of Esmeraldas is nearly three times higher than nationally [4]. Our study population may have a much lower socioeconomic status than the national one since Esmeraldas is one of the most economically deprived regions in the country and our study is conducted in isolated populations, which may strengthen the association between low socioeconomic status and a higher prevalence of diabetes and obesity [19–22]. Another reason for this higher prevalence could be the use of OGTT to diagnose T2DM, as the fasting plasma glucose test alone cannot detect almost 30% of cases [18]. These findings suggest that increased rates of NCDs as expected may occur in hard-to-reach rural areas, especially for women due to the high burden of overweight and obesity and improved access to unhealthy commodities such as sugary drinks and ultra-processed foods thanks to improved supply and aggressive marketing from big food and the lack of protective public health measures against commercial determinants of health [23]. In fact, our study shows a high prevalence of obesity among rural women; not only one out of two women between 40 and 64 years old have a BMI over 30, but also two out of five aged between 18 and 39. This finding is alarming as a young population with obesity and other risk factors will likely progress to develop diabetes and other chronic diseases associated with obesity as they age living in an obesogenic environment. The problem is even more concerning if we consider that these rural areas, with limited primary healthcare services focused on disease prevention, have also hindered access to secondary and tertiary/higher levels of healthcare services, only available in urban areas. The coming years will likely reveal increased mortality and comorbidities due to NCDs, with the following impact in an already impoverished socioeconomic setting.

However, while both men and women are exposed to the same context characteristics, the social and cultural norms differ. As shown in other studies, the employment characteristics of the sample shows how gender roles are strongly entrenched in rural areas, permeating the daily activities of men, who have the role of breadwinners, and women, who care for the family and household [24–28]. The double workload—productive and reproductive—affects women's ability to self-care and limits leisure time for physical activities [28, 29]. In addition, because of their role as caregivers, women in low-income areas tend to prioritise foods with higher nutritional value than the rest of the family and settle for remaining foods, which tend to have higher calorie content [30]. Over the last decades, road construction has improved the connectivity of rural areas, expanding the local infrastructure (internet, electricity…), but also unhealthy food, tobacco and alcohol. While the entire population is exposed to these risk factors, our data reveals a notable disparity in obesity rates between men and women. One of the possible reasons is that when unhealthy diets are ubiquitous, the role of physical activity becomes more important. While men continue to perform manual labour demanding intense physical activity, women's physical requirements have been facilitated by the advent of new technologies such as road transport, water pipes, household appliances and internet connexion, increasing sedentarism. This idea is reinforced by the fact that indigenous women, who still cultivate and harvest the fields, meet the WHO's physical activity recommendations in large proportions and have lower obesity rates. Finally, the high birth rate linked to young pregnancy and multiparity in these rural areas could also play an important role in the obesity prevalence due to postpartum weight retention [31–33]

Another interesting finding is the high proportion of people who eat an unhealthy diet. The low diversity of food groups, the low consumption of fruits and vegetables, the high consumption of processed foods, and industrially produced beverages high in refined sugar show a clear pattern of consumption associated with overweight, obesity and diabetes. The reasons for this type of diet are not only the lack of availability in the area and the high cost of buying fruits and vegetables but also resulting from an important cultural aspect. Ancestors subsisted on green plantain, yucca and proteins from hunting and fishing, and today a satiating diet high in carbohydrates and low in fruit and vegetable consumption remains predominant. The fact that men consume more vegetables could be explained by their improved access to them while working in the fields, while women tend to eat available foods in the household.

### Strengths and limitations

Our randomly selected population-based methodology provides the study with a representative sample of the Eloy Alfaro district as a major strength, along with around an 80% response rate thanks to the fieldwork experience of CECOMET, an organization of lay health promoters established in the region for more than 40 years. The COVID-19 pandemic influenced the study in several ways that could have affected the results: Firstly, the duration of results collection was longer than planned due to the movement restriction measures and protocol adjustment we carried out to prevent COVID infections. This increased the time lag between population census and data collection, which may have caused higher rates of displaced or deceased persons in our sample, along with migration to other regions to cope with the economic difficulties of the pandemic. Secondly, due to the impact of the pandemic on the economy of the population, information about household income could be affected, as well as physical activity and diet among other health behaviours [34–36]. While most of the data were collected between October 2020 and August 2021, data collection in the urban area of Borbón was interrupted to carry out in the rural part, which was only accessible by boat due to the temporary rise in rivers, and resumed in January 2022 to complete the sample.

In contrast with other studies, our sample did not detect differences in T2DM and obesity by socioeconomic characteristics (education, household income) [37–39]. One reason for this could be the homogeneity of the people living in this area where low incomes and low education are widespread. Furthermore, providing information regarding household income appeared to be a sensitive topic and some individuals were reluctant to reply, others may have underreported the family income when a portion of it came from government subsidies due to fear that it may be withdrawn. Furthermore, we collected information on household income and explored differences in diabetes and obesity among men and women, but a proportional distribution between partners cannot be assumed. Additionally, the low cases of obesity among men may have led to reduces statistical power and explain why differences according to sociodemographic characteristics were not detected.

These results may not be generalisable to a broader range of populations living in different rural areas, but they further support the idea of gender roles having a major impact on health inequities [40, 41]. Further research with this gender perspective is needed to better understand the mechanisms underlying obesity and diabetes in rural areas to design public health programmes that address health inequities in NCDs. Interventions should also focus on the prevention of obesity due to its high prevalence considering sex differences. These findings should help stakeholders to consider the sociodemographic characteristics of the population when designing interventions to reduce the prevalence of diabetes and its comorbidities according to sex and gender.

## Conclusions

Gender roles play an important role in the higher prevalence of obesity and diabetes in women compared to men, especially in hard-to-reach rural areas with a similar low socioeconomic status. Further research on the differential impact of gender roles on NCDs between men and women is needed. Gender-sensitive prevention and health promotion programmes should be implemented in order to reduce obesity and the burden of T2DM.

## Data Availability

The data used and/or analysed during the current study are available from the corresponding authors on reasonable request.

## Abbreviations

T2DM: Type 2 Diabetes
NCDs: Non-Communicable Diseases
LAC: Latin America and the Caribbean
OGTT: Oral Glucose Tolerance Test
BMI: Body Mass Index
OR: Odds Ratios
95%CI: 95% Confidence intervals
CECOMET: Centre of Community Epidemiology and Tropical Medicine
